# Overcoming the challenges of genome-editing essential genes

**DOI:** 10.1016/j.xpro.2026.104672

**Published:** 2026-07-02

**Authors:** Lydia Teboul, Benjamin Davies

**Affiliations:** 1 The Mary Lyon Centre, MRC Harwell, Didcot, Oxfordshire OX11 0RD, UK; 2 The Francis Crick Institute, London NW1 1AT, UK

**Keywords:** CRISPR, Genetics, Model Organisms, NMGN Focused Collection

## Abstract

This primer manuscript summarizes gene-editing strategies for limiting gene-editing activity with the aim of avoiding biallelic disruption. Mono-allelic editing of cells and embryos is advantageous when modeling dominant genetic disorders or when addressing essential or developmentally important genes, loss of function of which leads to severe phenotypes. Gene-editing reagents, such as the CRISPR-Cas9 system, are very efficient and frequently result in bi-allelic mutation of the selected target site. The article introduces different strategies for restricting editing to a single allele, exploring modifications to both the delivery of the gene-editing reagents and to the enzymes commonly used for gene editing themselves, along with design considerations for both the target sites and the repair template when trying to achieve knockin mutations.

## Graphical abstract

**Figure:**
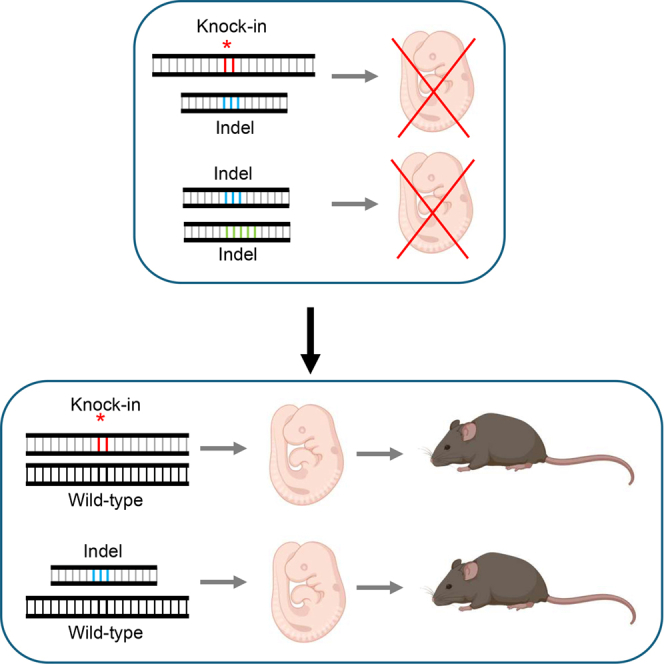

## Teaser

This primer manuscript summarizes gene-editing strategies for limiting gene-editing activity with the aim of avoiding biallelic disruption. Mono-allelic editing of cells and embryos is advantageous when modeling dominant genetic disorders or when addressing essential or developmentally important genes, loss of function of which leads to severe phenotypes. Gene-editing reagents, such as the CRISPR-Cas9 system, are very efficient and frequently result in bi-allelic mutation of the selected target site. The article explores different strategies for restricting editing to a single allele, exploring modifications to both the delivery of the gene-editing reagents and to the enzymes commonly used for gene editing themselves, along with design considerations for both the target sites and the repair template when trying to achieve knockin mutations.

## Introduction

Genetically modified mice are essential tools for studying gene function and modelling human disease. By introducing precise deletions, disease-associated mutations or by knocking-in functional sequences such as reporter genes, researchers can investigate normal cell biology and physiology, and replicate pathological conditions in vivo for therapeutic and diagnostic studies.

Site specific nucleases, especially CRISPR/Cas systems, allow rapid and efficient targeted mutagenesis in preimplantation mouse embryos.^1^^,^^2^ Typically, the CRISPR/Cas reagents, consisting of a Cas nuclease paired with a guide RNA that directs the nuclease to a specific genomic sequence, are delivered into the zygote by either microinjection^1^^,^^2^ or electroporation.^3^ Alternatively, delivery of CRISPR reagents to mouse zygotes has been achieved via recombinant virus^4^ or more recently via the application of viral-like particles.^5^ The Cas nuclease then generates a double strand break (DSB) at the target site and the cell rapidly repairs this break using its DNA repair machinery.^6^

The non homologous end joining (NHEJ)^7^ pathway often repairs the DSB without error, but repeated cutting by persistence of the Cas nuclease can lead to small insertions or deletions (indels) that can disrupt gene function, especially if they cause frameshifts or remove essential residues.^8^ Depending on the sequences adjacent to the DSB, the more error-prone microhomology-mediated end-joining (MMEJ) repair pathway may also play a role in mutagenesis at the site of the introduced DSB.^9^ Alternatively, when a template DNA molecule is also supplied, repair may proceed through homology directed repair^10^ (HDR) or single strand annealing,^11^ enabling precise copying of the template sequence into the genome. These mechanisms form the basis of CRISPR knockin strategies used to introduce point mutations, reporters, or other designed genetic changes.

High efficiencies of mutagenesis can now be achieved, with numerous projects achieving close to 100% bi-allelic target site mutation following the introduction of CRISPR/Cas ribonucleoprotein into the zygote (Table 1). Knockin efficiencies, in contrast, vary substantially, but several reports have shown, in particular with viral delivery of repair templates, efficiencies exceeding 50% (Table 1).^12^^,^^13^^,^^14^ One further complexity is the timing of the DSB repair relative to the cell division occurring after the gene editing reagents are introduced. In preimplantation embryos, persistence of the nuclease after cell division can make a mosaic outcome highly likely - that is, the resulting embryo consists of cells of many different genotypes and zygosities.^15^

**Table 1:** Approaches for gene editing and their reported efficiencies

Reference	Allele type generated	Editing approach	Founder efficiency	Target gene(s)
1	KO – indel (NHEJ)	Cas9 mRNA injection of embryos	1/7 mouse pups mutated	GFP
2	KO – indel; multiplex KO	Cas9 mRNA + sgRNA injection of embryos, **varying concentration of reagent**	∼50–90% founders mutated	*Tet1*, *Tet2*, *Tet3*, *Sry*, *Uty*
3	KO – indel (NHEJ)	Cas9 mRNA + sgRNA electroporation of embryos	0–100% edited founders	*Tet1*, *Tet2* and multiple other
29	KO – indel (NHEJ)	RNP electroporation	40–100% indel founders	*Cdh1*, *Cdk8*, *Kif11, Mecp2*
25	KO - Deletions	**Injection of one cell of a two-cell embryo**	∼15-25% KI founders, higher F0 viability	*Tyr, Gata4, Tet1 and Tet3*
25	KO – indel (NHEJ)	**Injection of one cell of a two-cell embryo**	∼10-75%	*Virma, Dpm1, Slc17a5, Ctla4*
26	Mono-allelic frameshift	**Pronuclear transplantation for parent-specific modification**	∼50-80%	*Anapc2, Peg10, Mash2*
21	KO - Deletions	Cas9 mRNA of RNP injection or electroporation	Variable; reduced for essentials	3313 genes
22	KI – reporter; conditional (HDR)	Cas9 mRNA + donor DNA piezzo injection of embryos	∼20–40% KI founders	*Nanog*, *Mecp2*, *Oct4*, *Sox2*
44	KI - Point mutation P KI (HDR)	**Cas9 mRNA + donor injection, interallelic donor (rat embryos)**	∼4–37% KI founders	*Tyr*, *Asp*, *Kit*
27	KI – cassette knockin (HDR)	Cas9 mRNA or RNP + circular donor	∼0–50% KI founders	*Cdx2*, *Actb*
29	KI - tag KI (HDR)	RNP electroporation + donor DNA	∼30% KI founders	*Tyr*, *Sox2*
50	KI – base editing (C→T)	**Cytosine base editor in 2-cell embryos**	∼45-100%	*Tyr*, *Dmd*
51	KI – base editing (A→G)	**Adenine base editor in 2-cell embryos**	∼70%	*Tyr*
12	KI - reporter or point mutation KI (HDR)	RNP electroporation + AAV donor	∼15-100% KI founders	*Rosa26 mouse and rat*
13	KI – cassette knockin (HDR)	AAV donor + RNP electroporation	∼18-00% KI founders	*Tyr*, *Sox2*, *Rosa26*
24	KI – allele-specific (HDR)	**Cas9 mRNA with optional donor cytoplasmic injection in heterozygous embryos**	∼30%	*Crygc*
56	KI – precise prime-edited alleles	**Prime editing in 2-cell embryos**	∼1-80% edited founders, target dependent	*Rnf2*, *Chd2*, and multiple others
14	KI – cassette knockin (HDR)	RNP electroporation + AAV donor	∼0-80% KI founders	Multiple loci
60	KI – reporter gene fusion (HDR)	**Cytoplasmic injection and repair pathway inhibitors**	∼6-96% KI founders, other allele types inhibited	*Sod1*, *Slbp, Cenpe*, Grin2a, Trio
39	KI - Point mutation P KI (HDR)	**dCas9-Cas9 combination RNP + donor electroporation**	∼0-54% KI founders, higher F0 viability	11 genes

The table summarizes published variations of protocols aimed at generating genetically modified alleles (KO – Knockout; KI – Knockin) and their relative efficiencies, expressed as the proportion of founders carrying the desired mutation. Where the application of the modified approach led to the generation of desired founders (where conventional approaches failed), the approach is listed in bold.

**Figure 1:**
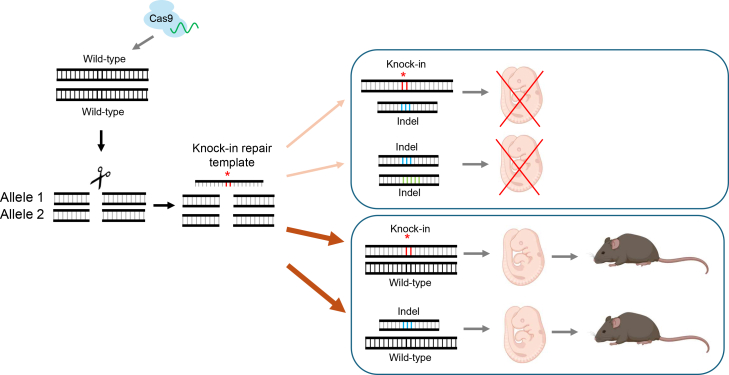
CRISPR/Cas mutagenesis of essential genes leads to high levels of mutagenesis Methods which encourage heterozygous targeting and the preservation of a wild-type copy of the gene can greatly assist the production of both Knockout and Knockin alleles in essential genes.

In establishing lines of genetically modified animals, the downside of this high efficiency of mutagenesis becomes apparent when addressing genes or sequences whose role is essential for normal cellular physiology and development. Studies in mouse have suggested loss-of-function of approximately a third of the protein coding genome can impact viability.^16^ In these cases, the high efficiency of mutagenesis, although achieving the desired mutagenesis event, results in offspring with high levels of bi-allelic mutagenesis and lethality is common (Figure 1). Where embryonic lethality occurs in the majority of embryos, the pregnancy of the recipient female is frequently lost, and very few live pups are recovered, complicating the production of such a model. Furthermore, where the target gene has a critical role at later postnatal stages of development or in adulthood, substantive phenotypes compromising the welfare of the mouse^17^ or its ability to breed may occur,^18^ preventing the establishment of lines.

This primer explores ways to tackle these issues in an attempt to reduce the on-site damage caused by the CRISPR/Cas reagents or to restrict mutagenesis to a single allele. A heterozygous editing outcome is of considerable importance to avoid viability issues when exploring the function of developmentally essential genes and for accurate modelling of human dominant mutations. In addition, many of the approaches could be useful for allele-specific therapeutic gene editing.

Similar challenges relating to bi-allelic mutagenesis of essential genes are also encountered when applying gene editing reagents in cultured cells.^19^ If the target gene is essential for cell viability or division, the desired models are simply not produced in culture.^20^ Although this review focusses on the editing of mouse models, many of the principles outlined are appropriate and have been informed by in vitro work with cells, in particular stem cells.

## Methods to control mutagenesis of essential genes

The mutagenesis and the establishment of genetically modified lines for genes which manifest a cellular, developmental, fertility or severe postnatal phenotype is challenging due to the high mutagenic activity of the site-specific nuclease when introduced into the developing zygote. Large consortium projects aimed at knocking-out all mouse genes have also concluded that the essentiality of the target gene has the most influence on project success.^21^

For directed change of the genome, for example, for the knockin of a disease-related pathogenic SNP, repair templates are added with the CRISPR/Cas reagents as a standard method.^22^ With NHEJ repair typically being dominant over templated repair mechanisms,^6^ a likely outcome is the production of compound heterozygous founder mice harbouring the desired knockin SNP on one allele and an indel mutation on the other allele. When the mutation to be introduced is likely to cause an overt phenotype (as is often the case when modelling human genetic disease) and the target gene has an essential function, such a compound heterozygous mouse could also lead to embryonic lethality or a manifestation of an undesirable phenotype that precludes onward breeding.

Here we list and comment on various strategies and design considerations which can be employed to try to tackle these issues, reviewing the different steps of the mutagenesis process that can be adapted to achieve mono-allelic mutagenesis.

### Methods for the delivery of gene-editing reagents

1. Gene targeting in embryonic stem cells

The traditional approach for generating mouse models has involved gene targeting in embryonic stem cells (ES), which are then injected into blastocysts to generate chimeras.^23^ In these experiments it is feasible to generate and screen many cell clones, with a high likelihood of identifying and validating heterozygous mutations. In comparison with direct zygote manipulations, this is a laborious, lengthy process that relies on the quality of parental ES cells and much technical expertise, but it can be used to ensure that mutations are kept in a heterozygous state throughout the genome engineering process.

2. Manipulating the 2-cell embryo

Conventionally, gene editing reagents are introduced into the one-cell fertilized zygote as this provides the most direct way of generating a founder mouse in which the desired mutation is incorporated into the target gene. Editing efficiencies for simple knockout frequently approach 100%, whereas for knockin alleles, a lower rate of between 5-50% is generally observed (Table 1). Given these high efficiencies of mutagenesis, when addressing essential genes, this strategy has the highest chance of the manifestation of a disadvantageous phenotype which might preclude onward breeding or even developmental progression and viability of the founder.

Injecting into one blastomere of the 2-cell embryo is an approach which has been shown to overcome these effects in certain cases.^24^^,^^25^ Manipulating a single blastomere effectively forces mosaicism of the resulting embryo, tempering detrimental effects which might contribute to developmental arrest. The overall impact on efficiency is minor, with high rates of knockout and knockin allele production reported (Table 1). The success of this strategy is more likely in the case of cell autonomous phenotypes as an appreciable number of non-mutated cells will compensate for the potential impact of bi-allelic mutant cells present within the mosaic founder.

From a welfare perspective, it is still unclear whether certain substantive phenotypes could manifest in a mosaic mouse, so although this method can certainly help the development and birth of putative engineered founders, a case-by-case welfare assessment on the impact of target gene loss-of-function in adult mice is critical.

3. Pronuclear transplantation

An interesting approach to generating allele-specific manipulations has been reported which takes advantage of pronuclear transplantation techniques.^26^ Gene editing reagents are injected into mouse MII oocytes at low concentrations, which are then fertilized in vitro. A resulting pronuclei, presumably harbouring a single mutant target gene, can then be transplanted into enucleated oocytes and used for live rederivation. Reported efficiencies of generating knockout alleles were high (50-80%, Table 1). When addressing the essential *Anapc2* gene, loss of function of which results in early embryonic lethality, heterozygous knockouts were recovered when using this method. In contrast, conventional approaches, delivering gene editing reagents for *Anapc2* in the fertilized zygote, resulted in preimplantation developmental arrest.^26^ Although this approach did overcome the genome engineering challenge caused by the bi-allelic loss-of-function phenotype, it does require considerable embryo manipulation expertise that is rare and might represent a significant challenge for laboratories engineering such mutations to acquire.

### Reducing the activity of genome-editing reagents

1. Lower concentration of reagents

To reduce the mutagenic effects of CRISPR, several studies have investigated the effect of lowering the concentration of reagents and have found overall mutagenesis can be reduced.^27^ For example, reducing the concentration on RNP from 100 μg/μl to 30 μg/μl reduced the mutagenesis efficiency at the *Actb* locus from 50% to 15.4%^27^ (Table 1). This overall reduction in mutagenesis rate has been shown to lead to a decrease in bi-allelic targeting (for example from 83% to 67% at the *Tyr* locus^28^ and from 82% to 57% at the *Tet1* locus^2^ (Table 1)), and thus a reduction in reagent concentration would be beneficial when addressing genes loss-of-function of which leads to embryonic lethality or a substantive phenotype. However, as the efficiency of genome editing remains generally unpredictable,^29^ greatly depending upon the genetic context and the mode of delivery, an iterative approach may be required for each gene edit to determine an appropriate titration of activity.

2. Use of nickases

Limiting the introduction of DSBs may be key to tempering the activity of mutagenesis occurring at the CRISPR/Cas target site. Site specific nucleases can be engineered to only address one of the two strands of DNA, creating enzymes known as nickases.^30^ Early studies in human cells with zinc-finger nickases revealed that DNA nicks can be repaired by introduced templates, albeit at lower efficiency than the fully active nuclease (between 12-50% reduction observed).^31^^,^^32^^,^^33^ Interestingly, a reduction in NHEJ-induced indels at the target site was seen in some of these studies, suggesting this method could be effective at reducing the onsite mutagenesis that complicates genetic engineering of essential genes. Although paired nickases to induce DSBs have been used for targeted mutagenesis in mouse zygotes,^34^ we are not aware of an example where a single nick-induced templated repair achieves founder production.

3. Use of catalytically inactive Cas9

The two catalytic domains of the Cas9 nuclease can be silenced by simple point mutations (D10A, H884A)^30^ and the resulting inactive nuclease is known as deactivated-Cas9 (dCas9). dCas9, when complexed to a specific sgRNA, results in site-specific binding to the genome, but no DNA cleavage occurs. This property was originally applied to transcribed regions of the genome to achieve steric inhibition of the RNA polymerase, reducing expression in a method known as CRISPR-interference.^35^ dCas9 binding to the genome near an introduced DSB was also shown to inhibit end resection.^36^ The same principle of dCas9 binding to the genome can also be used to protect an allele from an active Cas9. Subsequently diluting the active CRISPR reagents with dCas9 complexed with sgRNA leads to a reduction in bi-allelic mutation and, in the case of essential genes, a larger proportion of viable heterozygous mutated mice can develop to term and thus be used to establish lines of genetically modified mice.^37^^,^^38^ Editing efficiencies of up to 54% have been reported for this method in mouse zygotes^38^ (Table 1). A limitation of this approach is that it may not be applicable to experimental designs that require two DSBs such as the generation of floxed exons for conditional mutagenesis but interestingly, the approach may help to prevent off-target nuclease activity. The use of this strategy is in its early days, and more examples of its use will be required to appreciate its applicability.

### Design of CRISPR target sites and templates

1. Distancing the SNP change from the DSB site

The issues emerging with genetic engineering in zygotes also apply in vitro, for example when engineering disease-associated SNPs into stem cells. The high activity of the CRISPR/Cas system can lead to SNP installation on one allele in combination with indel mutations on the other. Achieving purely heterozygous cells, which is frequently the goal of modelling human genetic disease, can be challenging.

A method, known as CORRECT, has been established in human iPS cells which uses two approaches to increase the likelihood of installing heterozygous mutations.^39^ Firstly, it is well established that mutations in the protospacer or the protospacer adjacent motif, which are designed with synonymous codon changes when the target site lies within a coding exon, can reduce unwanted indel mutations and, in yeast, has been shown to help achieve mono-allelic targeting.^40^ Secondly, the method takes advantage of the inverse relationship between the rate of incorporation of a SNP and the distance this mutation lies from the DSB. Purely heterozygous mutations can be favoured by increasing the distance between the SNP change and the DSB site. This method was applied successfully to introduce heterozygous mutations associated with Alzheimer’s disease in human iPS cells.^39^

Although this approach has never been systematically tested in a similar manner in mouse zygotes, the design principles would be very applicable to mouse engineering, when trying to generate heterozygous knockin mice and potentially avoiding the manifestation of phenotypes in the founder generation.

2. Mutagenesis of hybrid backgrounds

Allele-specific targeting using CRISPR/Cas reagents which discriminate between mutant and wild-type alleles in heterozygous mouse zygotes has been demonstrated in several studies.^41^^,^^42^ These studies confirm that single nucleotide differences between alleles can allow selective mutagenesis of only one allelic copy. SNP differences between inbred strains could be exploited to allow mono-allelic targeting when CRISPR reagents are introduced into hybrid embryos. This has been achieved in rat, where selective mutagenesis of F344 Tyrosinase allele was achieved in hybrid (F344 x DA)F1 zygotes.^43^ The availability of such polymorphic sites across a target gene may limit the application of this approach, and indeed the tolerance of the nuclease for certain mismatches may also influence the degree to which the two alleles can be discriminated.

3. Intronic dual cutting strategy

**Figure 2:**
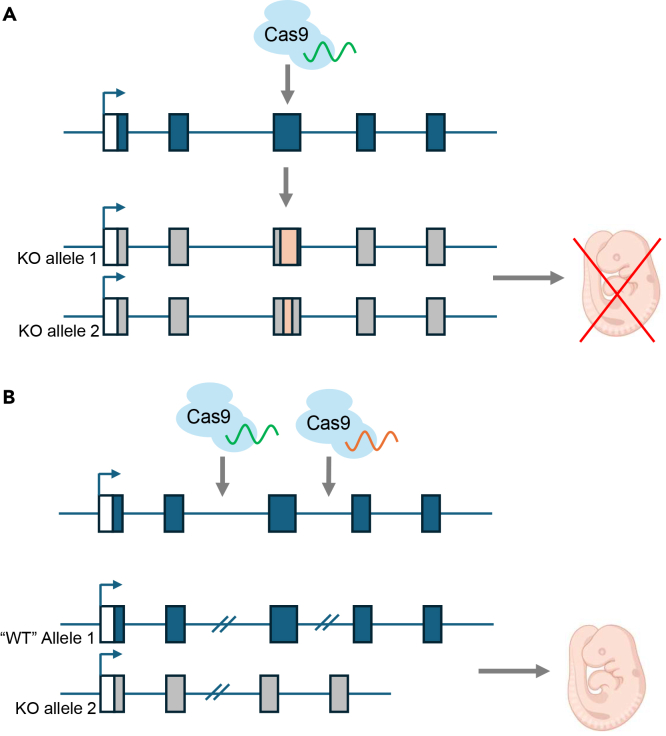
Site-specific nucleases targeting intronic regions (A) CRISPR/Cas mutagenesis of a coding exon can lead to high rates of bi-allelic mutation, causing lethality when targeting essential genes. (B) Changing the strategy to excise genomic sequence by targeting CRISPR/Cas to introns can decrease the overall mutagenesis levels, improving mouse production efficiency and allowing the desired loss-of-function allele to be generated.

To disrupt gene function, site-specific nucleases are often directed to exonic regions. The indels generated during repair can shift the open reading frame, creating nonsense transcripts that either produce nonfunctional proteins or trigger nonsense-mediated decay (Figure 2A). Alternatively, two nucleases can be targeted to different regions of the same gene *in cis*, allowing non-homologous end joining (NHEJ) repair of the two DSBs to delete or invert the intervening sequence.^44^^,^^45^ More commonly, however, each DSB is repaired independently, resulting in separate indel mutations at each site.

For essential genes, targeting DSBs to intronic regions - sufficiently far from splice junctions - minimizes gene disruption, as the small intronic indels most likely preserve normal gene and protein expression (Figure 2B). This can increase the proportion of embryos that develop successfully after editing. At a lower but still meaningful frequency, an *in cis* deletion between the two intronic cut sites will occur, removing part of the coding sequence and thereby disrupting gene function. Although not systematically explored for the mutagenesis of essential genes, this strategy would be expected to decrease the number of mice harbouring lethal loss-of-function alleles and thus improve the likelihood of obtaining a compound heterozygous mouse carrying both the desired deletion allele and a benign allele containing only simple intronic indels.

Mutagenesis of non-coding regions, however, carries the risk of disrupting gene splicing, particularly when the introns being addressed are under 100 bp in size or where the introns lack canonical characteristics, making it challenging to avoid functional elements. In this context, it is interesting to note that splice site prediction using machine learning algorthms are now improving prediction of essential splicing elements which may help reduce the risk.^46^

4. Use of intronic CRISPR sites for gene knockin

**Figure 3:**
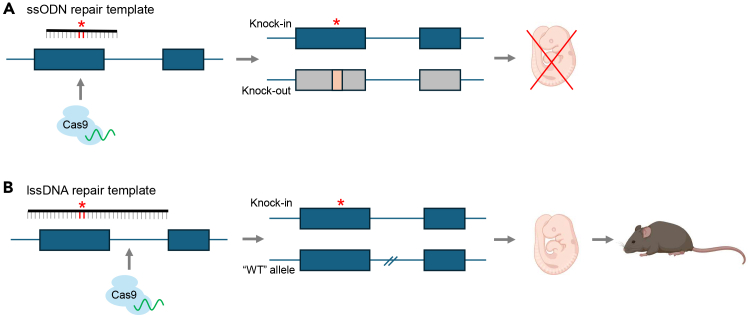
Achieving point mutations near the extremities of exons by moving the CRISPR/Cas target site to intronic sequences (A) Typically, a CRISPR/Cas target site very close to the desired mutation is chosen to maximize successful repair with the ssODN template. The high frequency of bi-allelic indel mutation within the reading frame could lead to lethality when targeting essential genes. (B) Moving the CRISPR/Cas target site to non-functional, non-coding intronic sequence in combination with the use of a longer repair template may help reduce the mutagenic burden and facilitate production of the desired mutant.

As explained above, generating a line of mice with a knockin mutation within an allele of an essential gene is complicated by an accompanying loss-of-function indel mutation on the other allele (Figure 3A). Where the mutation to be engineered into the genome lies close to an intron/exon boundary, one method of reducing damage to the coding genome is to position the CRISPR/Cas target site in the intron. As described above, assuming the CRISPR/Cas target site is positioned away from splicing machinery and other functional sequences, this can reduce disruption of the target gene as an accompanying indel mutation would not be coding. In combination with longer repair templates, such as long-single-stranded DNA, this approach can then be used to incorporate the desired point mutation (Figure 3B).

We have explored the use of this approach when targeting genes, loss-of-function of which leads to embryonic lethality. By directly comparing this method with a conventional approach using cutting the exons at the SNP site and using an ssODN repair template, the intron-shifted approach led to a significant increase in pregnancy (Table S1; Fisher exact test *P* = 0.0008) following the embryo transfer of gene edited embryos and the production of live pups (Table S1; Fisher exact test *P* < 0.0001), allowing the completion of the projects successfully. This approach would likely lead to site damage within the intron and subsequently it does rely on a complete functional annotation of the adjacent non-coding sequence to avoid unintended effects of potential indels left after DSB repair, but as mentioned above splice site prediction is continually improving.^46^

5. Use of wild-type repair templates

**Figure 4:**
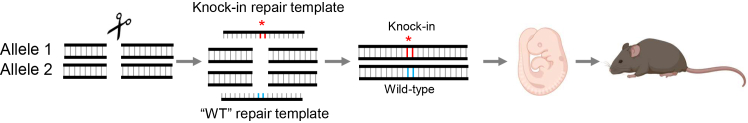
Co-introduction of both mutant and wild-type repair templates allows the repair of the CRISPR/Cas-induced double strand break with either of the introduced templates When both alleles are repaired by homologous directed repair, the use of a wild-type template could increase the probability of generating heterozygous founder mice, avoiding the potential lethality which might result from the homozygous installation of a mutation within an essential gene.

When performing knockin mutations using repair template, one way of encouraging the production of heterozygous editing is to use a mix of repair templates – one of which encodes the desired mutation and the other is simply a wild-type copy of the gene.^47^ Originally reported in iPS cell editing, repair of the CRISPR/Cas-induced DSB can then occur with either template, and when homozygous templated-repair occurs, iPS cell clones can be recovered which are heterozygous for the mutation of interest. Both templates incorporate silent protospacer blocking mutations to prevent recutting of the correctly repaired allele (Figure 4). The approach however relies upon bi-allelic templated repair, which represent the less frequent repair pathways active within the cell – although feasible, the desired outcome most probably will not be achieved at high frequency.

### Alternative CRISPR-based editing approaches

Combinations of site-specific DNA nickase or dCas9 with effector molecules with enzymatic domains have been used to great effect for achieving targeted enzymatic changes to the genome. Further development of these enzyme systems is aimed at improving their accuracy in the context of therapeutic editing. As new enzymes with reduced indel activity become available, they will be highly useful for avoiding the generation of unwanted loss-of-function alleles that might compromise the generation of animal models.

1. Base editing

In base editing, the enzymatic domains are typically deaminases which act on the nucleotide bases in the proximity of the DNA nick and can be used for A-to-G and C-to-T base transitions.^48^ These enzymes have been applied in mouse zygotes by microinjection for the production of knockin mouse models.^49^^,^^50^ Efficiencies were generally high, varying between 45% and 100% (Table 1), but clearly this technology is only appropriate for a subset of SNP Knockin mutations. However, this method is relevant for mono-allelic targeting of essential genes as the rate of indel mutagenesis occurring at the target site is typically low. In vitro studies with cytosine and adenine base editors have achieved indel rates of less than 1%.^51^^,^^52^^,^^53^ However, such low incidence of indels is not always achieved^49^ and successful application of these enzymes is highly dependent on careful design.

2. Prime editing

Prime editing technology comprises a Cas9 nickase fused to a reverse transcriptase domain, with the information to be introduced into the genome encoded as a 3′ extension of the guide-RNA used for the targeting.^54^ Prime editors have been successfully delivered to zygotes for the purpose of targeted genomic change and high efficiencies of mutagenesis up to 80% have been observed (Table 1), with an absence of indel mutations reported.^55^ This is consistent with the low (0.60 ± 0.17%) rates of indel mutations reported when these editors are used in vitro.^56^ Again, each design must be validated in the laboratory as current design software is not entirely predictive.^57^ This technology in mouse zygotes has been validated for relatively small changes within the genome (typically alteration of a few bases),^55^ however prime-editing is now being used effectively for large scale manipulations of the genome^58^ which might suggest larger edits may become feasible for mouse line engineering as the technology becomes established.

## Conclusion

Restricting editing activity to a single allele is a desirable outcome when trying to model heterozygous genetic mutation in model systems and when addressing genes that are essential to life and reproduction or are associated with welfare issues. As outlined in this primer, all aspects of the gene editing pipeline can be modified to try to achieve high rates of mono-allelic targeting and to reduce the potential negative effects of bi-allelic mutagenesis. Considerations of the delivery and modifications to the gene editing nuclease being used are important – concentration of reagents, use of a nuclease targeting only one strand of target DNA and the inclusion of catalytically inactive nuclease are all approaches which can be used in parallel to temper the activity of the gene editing reagents. In addition, the actual design of the knockout or knockin allele is important, both in consideration of where the nuclease will introduce DSBs and the repair template used when knockin mutations are required. Targeting the nuclease to intronic sequences may be helpful in reducing the functional consequences of mutagenic activity on an essential gene, and the inclusion of wild-type repair templates to introduce mutant but benign alleles may also be a feasible strategy. For any specific edit, it is likely that a combination of approaches may be useful for achieving manageable rates of mono-allelic mutagenesis.

With respect to the production of knockin alleles, experimental approaches using inhibitors of the MMEJ/NHEJ machinery have been reported^59^ which help shift the balance of the DNA repair pathways operating in the cell to increase the efficiency of template-driven repair. Similarly, machine learning algorithms are now able, to a certain extent, to predict the prominent NHEJ/MMEJ-mediated repair outcomes following CRISPR/Cas-induced DSBs based on the primary target sequence.^60^ Such tools could, in theory, help avoid the use of sgRNAs likely causing significant out-of-frame mutations, in an effort to reduce undesirable mutagenesis when trying to install point mutations in coding exons of essential genes. Whether these methods would also help reduce the occurrence of bi-allelic mutation and the associated problems when targeting essential genes within the mouse zygote remains unclear but could be the focus of additional investigations. Artificial Intelligence led protein evolution approaches are also being used to improve and adapt the characteristics of gene editing machinery which could reduce the incidence of potentially disruptive indel mutations.^61^

Importantly, despite all the above considerations, it is well established that there are haplo-insufficient genes where mono-allelic mutations are lethal or associated with severe defects.^19^ Genetic perturbation of such genes would necessitate more complex conditional allele design. Nonetheless, the approaches summarized in this primer would assist in the production of such models by reducing mutagenic loss-of-function events.

## Acknowledgments

B.D. is supported by the 10.13039/100010438Francis Crick Institute (which receives its core funding from 10.13039/501100000289Cancer Research UK [CC1111], the UK 10.13039/501100000265MRC [CC1111], and the 10.13039/100010269Wellcome Trust [CC1111]) and the 10.13039/501100000265Medical Research Council National Mouse Genetics Network (MC_PC_21048). L.T. is supported by 10.13039/100014013UKRI/10.13039/501100000265MRC funding through awards MC_UP_2201/1, MC_UP_2201/2 and MC_UP_2201/3.

The authors thank Daniel Biggs, Chris Preece, and Samy Alghadban for primary data and Graham Duddy for critical reading of the manuscript.

## Author contributions

L.T. and B.D. co-wrote and edited the primer.

## Declaration of interests

The authors declare no competing interests.

## Supplemental information

Table S1. Production efficiencies of mouse C57BL/6J zygotes electroporated with CRISPR/Cas9 targeting either an exonic or intronic region of two essential genes
