# Supplementary material for: Overcoming the challenges of genome-editing essential genes
Source: STAR Protoc. 2026 Jul 2;7(3):104672. doi: 10.1016/j.xpro.2026.104672 (PMC13352379; doi:10.1016/j.xpro.2026.104672)
Supplement: Table S1. Production efficiencies of mouse C57BL/6J zygotes electroporated with CRISPR/Cas9 targeting either an exonic or intronic region of two essential genes [file mmc1.pdf]

Gene	Design	sgRNA	No. of transfers	No. pregnant	% Pregnant	No. of embryos transferred	No. of pups born	% birth rate
Tpm1	Exonic	GAGCGACCTGGAACGTGCAG	3	1	33%	88	0	0.0%
Tpm1	Intronic	GACTAAACAGCATGACCTTC	7	5	71%	205	25	12.2%
Hnf4a	Exonic	ACGGCTGCAAGGGGTTCTTC	15	2	13%	447	5	1.1%
Hnf4a	Intronic	GAAGGGCAGATGCCTCAAGA	15	9	60%	369	32	8.7%

Table 1 – Production efficiencies of mouse C57BL/6J zygotes electroporated with CRISPR/Cas9 targeting either an exonic or intronic region of two essential genes. The pregnancy rate resulting from embryo transfer of manipulated zygotes and the birth rate, expressed as a percentage of the total number of embryos transferred, is shown. Both metrics are increased significantly by intronic targeting (% Pregnancy: Fisher exact test $P = 0.0008$; % Birth rate: Fisher exact test $P < 0.0001$)
